# Rabeprazole exhibits antiproliferative effects on human gastric cancer cell lines

**DOI:** 10.3892/ol.2014.2354

**Published:** 2014-07-16

**Authors:** MENGLI GU, YAN ZHANG, XINXIN ZHOU, HAN MA, HANGPING YAO, FENG JI

**Affiliations:** 1 Department of Gastroenterology, The First Affiliated Hospital of Medical College of Zhejiang University, Hangzhou, Zhejiang, P.R. China; 2 State Key Laboratory for Diagnosis and Treatment of Infectious Diseases, The First Affiliated Hospital of Medical College of Zhejiang University, Hangzhou, Zhejiang, P.R. China

**Keywords:** extracellular signal-regulated protein kinase 1/2, proton pump inhibitors, gastric cancer, antineoplastic therapy, rabeprazole

## Abstract

Intracellular proton extrusion in gastric cancer cells has been reported to promote cancer cell survival under acidic conditions via hydrogen/potassium adenosine triphosphatase (H^+^/K^+^-ATPase). Rabeprazole is a frequently used second-generation proton pump inhibitor (PPI) that irreversibly inactivates gastric H^+^/K^+^-ATPase. Therefore, we hypothesized that rabeprazole could reduce the viability of gastric cancer cells. In the present study, four human gastric cancer cell lines and one non-cancer gastric cell line were cultured. Cell viability, the α- and β-subunits of H^+^/K^+^-ATPase and cellular apoptosis were analyzed by dye exclusion assay, reverse transcription-polymerase chain reaction and annexin V-fluorescein isothiocyanate/propidium iodide staining, respectively. The expression level of total extracellular signal-regulated protein kinase 1/2 (ERK 1/2) and phosphorylated-ERK protein was detected by western blot analysis. Gastric cancer cell lines were more tolerant of the acidic culture media than non-cancer cells. Administration of rabeprazole led to a marked decrease in the viability of MKN-28 cells. Exposure to rabeprazole induced significant apoptosis in AGS cells. Rabeprazole completely inhibited the phosphorylation of ERK 1/2 in the MKN-28 cells, whereas the same effect was not observed in either the KATO III or MKN-45 cells. The ERK 1/2 inhibitor, PD98059, attenuated the viability of the AGS cells. A similar antiproliferative effect was observed in the rabeprazole treatment group. In addition, PD98059 and rabeprazole were able to efficaciously inhibit the phosphorylation of ERK 1/2 in the gastric cancer cells. Therefore, it was concluded that rabeprazole can attenuate the cell viability of human gastric cancer cells through inactivation of the ERK1/2 signaling pathway. The results of the present study demonstrate that rabeprazole inhibits the viability of gastric cancer cells *in vitro* and may serve as a novel antineoplastic agent.

## Introduction

Hydrogen/potassium adenosine triphosphatase (H^+^/K^+^-ATPase), normally contained within the lumen of gastric parietal cells, plays a vital role in the maintenance of cellular pH homeostasis by exchanging luminal K^+^ for cytoplasmic H^+^. This particular proton pump also participates in the formation of the abnormal pH gradients that are typical of gastric cancer cells during tumorigenesis (1). H^+^/K^+^-ATPase is composed of a 114-kDa α-subunit and a 35-kDa β-subunit. The α-subunit functions as a catalyst and a transporter of the proton pump, while the β-subunit is responsible for endocytic retrieval of the complex from the canaliculus (2).

Proton pump inhibitors (PPIs), substituted 2-pyridyl-methyl- sulfinyl-benzimidazole derivatives, exert their anti-secretory effects through the inhibition of gastric H^+^/K^+^-ATPase (3). PPIs are prodrugs that require protonation in acidic conditions for full activation (4). In their acid-activated form, PPIs bind covalently through disulfide bonds between cysteine residues located in the luminal vestibule of the proton pumps, leading to irreversible inhibition of the gastric proton pumps (5). Rabeprazole is a second-generation PPI that inactivates the gastric pump through covalent binding, causing a rapid and sustained inhibition of intracellular proton efflux, as well as raising the extracellular pH (6,7).

As gastric cancer cells *in vivo* often exist in an ischemic microenvironment with acidic conditions, it is of great importance to maintain cellular pH homeostasis for the function and survival of cancer cells (8,9). The acidified microenvironment in tumors is a consequence of the production of acidic by-products from rapid and large amounts of glycolysis (10,11). To avoid the intracellular accumulation of acidic moles, otherwise detrimental to cell survival, cancer cells enhance their ability to eliminate intracellular protons (12,13). Thus, intracellular proton extrusions in gastric cancer cells can promote cancer cell survival under acidic conditions. However, this protective mechanism can be inhibited by PPIs. PPIs are able to convert into the active form under hypoxic and acidic conditions in gastric cancer cells, a result of the upregulated anaerobic glucose metabolism. PPIs target gastric cancer cells and disturb cellular pH homeostasis. Previous studies have indicated that gastric cancer cells are more vulnerable to cell death than non-cancer cells following PPI treatment (14).

Taken together, these data show that PPIs may target gastric cancer cells and exert their antineoplastic effects locally by taking advantage of the low extracellular pH of gastric cancers, as a target and a way to specifically activate drugs within the tumor tissues. The present study investigated whether rabeprazole could exert an antineoplastic effect on gastric cancer cells and analyzed the possible anticancer mechanism of rabeprazole.

## Materials and methods

### Cell culture and reagents

Human gastric cancer cell lines, AGS, KATO III, MKN-28 and MKN-45, were purchased from the Shanghai Institute of Digestive Disease (Shanghai, China). The gastric cancer cell lines were cultured in RPMI 1640 medium (Gibco BRL, Grand Island, NY, USA) with 10% fetal bovine serum and 100 U/ml penicillin. The non-tumorigenic human gastric epithelial cell line, GES-1, was established from fetal stomach cells infected with the SV40 virus (15). The GES-1 cells were grown in DMEM with 10% fetal bovine serum. These cells were maintained in a humidified incubator at 37°C in a 5% CO_2_ atmosphere.

Rabeprazole (H20020330) was obtained from Jiangsu Hansoh Pharmaceutical Co., Ltd. (Jiangsu, China). The ERK 1/2 inhibitor, PD98059, was purchased from Selleck Chemicals LLC (Shanghai, China).

### Analysis of cell viability

To determine the effect of acidic media on cell viability, three gastric cancer cell lines, KATO III, MKN-28 and MKN-45, and one control human gastric cell line, GES-1, were cultured in media with various pH levels (7.5, 6.5 and 5.5) for 24 h. The AGS cells were further cultured at various pH levels (7.4, 6.4, and 5.4) for 16 h following treatment with rabeprazole and PD98059 for 2 h, respectively. The cell viability was determined by a dye exclusion assay. The viability percentage was calculated using the following formula: The number of viable cells counted (unstained cells) / the number of total cells × 100.

### Reverse transcription polymerase chain reaction (RT-PCR) of α- and β-subunits of H^+^/K^+^-ATPase

Total RNA from gastric cancer cell lines was extracted using the TRIzol reagent (Beyotime Institute of Biotechnology, Shanghai, China) according to the manufacturer’s instructions. The RT reaction for the first-strand cDNA synthesis was carried out with reverse transcriptase (Beyotime Institute of Biotechnology) using 2 μg total RNA. Specific primers were as follows: Human H+/K+-ATPase α-subunit forward, 5′-TCT CTC CGA GCA GCG CA-3′ and reverse, 5′-CGT CGC CAC TCT TGC TGT CG-3′; human H+/K+-ATPase β-subunit forward, 5′-ATG GCG GCT CTG CAG GAG AA-3′ and reverse, 5′-CGT GGA GAC TCT GTG TGA CG-3′; human GAPDH forward, 5′-AGG TCG GAG TCA ACG GAT TTG -3′ and reverse, 5′-GTG ATG GCA TGG ACT GTG GT-3′. RT-PCR was performed using the Premix Ex Taq kit (Takara Biotechnology (Dalian) Co., Ltd., Dalian, China). The amplifications were performed in 50-μl reaction volumes with an initial denaturation at 94°C for 5 min prior to 38 thermal cycles of 94°C for 1 min, 55°C for 30 sec and 72°C for 1 min, with a final extension at 72°C for 10 min. The amplified PCR products were subjected to electrophoresis in 1% agarose gels at 80 V for 30 min and visualized by ethidium bromide staining.

### Annexin V-fluorescein isothiocyanate (FITC)/propidium iodide (PI) staining

Induction of apoptosis was detected by an annexin V-FITC/PI apoptosis assay kit (Major BioTech Co., Ltd., Shanghai, China), according to the manufacturer’s instructions. Treated and control cancer AGS cells were harvested following trypsinization and washed twice with cold phosphate-buffered saline (PBS). The cells were centrifuged at 500 × g for 5 min, and the supernatant was removed. The suspensions were incubated with 20 ml annexin V-FITC and 20 ml PI for 15 min, at room temperature and in the dark. The cells were then evaluated by flow cytometry (Beckman Coulter, Inc., Miami, FL, USA) and the data were analyzed using WinMDI 2.9 (Purdue University Cytometry Laboratories, West Lafayette, IN, USA). FITC-positive cells were classed as early apoptotic cells and FITC- and PI-double positive cells were interpreted as late apoptotic cells. All experiments were conducted in triplicate.

### Western blot analysis

The treated and control cells were harvested, washed with cold PBS and lysed in cold lysis buffer. Subsequent to incubation on ice for 30 min, lysates were centrifuged at 12,000 × g for 10 min at 4°C, and supernatants were then collected. Protein concentrations were determined by the Bicinchoninic Acid Protein Assay kit (Pierce, Rockford, IL, USA), following the manufacturer’s instructions. Samples containing 50 μg protein were electrophoresed on 12% sodium dodecyl sulphate-polyacrylamide gels, and transferred to polyvinylidene difluoride (PVDF) membranes using a semidry transfer system (Bio-Rad Laboratories, Hercules, CA, USA). The PVDF membranes were blocked with a 5% bovine serum albumin and Tris-buffered saline with Tween^®^ 20 buffer for 2 h at room temperature and incubated with specific polyclonal rabbit anti-human antibodies corresponding to phosphorylated-ERK and total ERK (Cell Signaling Technology, Beverly, MA, USA) overnight at 4°C.

### Statistical analysis

Results are expressed as the mean ± standard deviation. The apoptotic rates in the rabeprazole and control groups were compared by Student’s t-test. One-way analysis of variance was performed for multiple comparisons and the statistical significance between the groups was determined by Duncan’s multiple range test. P<0.05 was considered to indicate a statistically significant difference. All analyses were performed using SPSS v15.0 (SPSS, Inc., Chicago, IL, USA).

## Results

### Gastric cancer cells show more tolerance to acidic culture media than non-cancer cells

**Figure 1:**
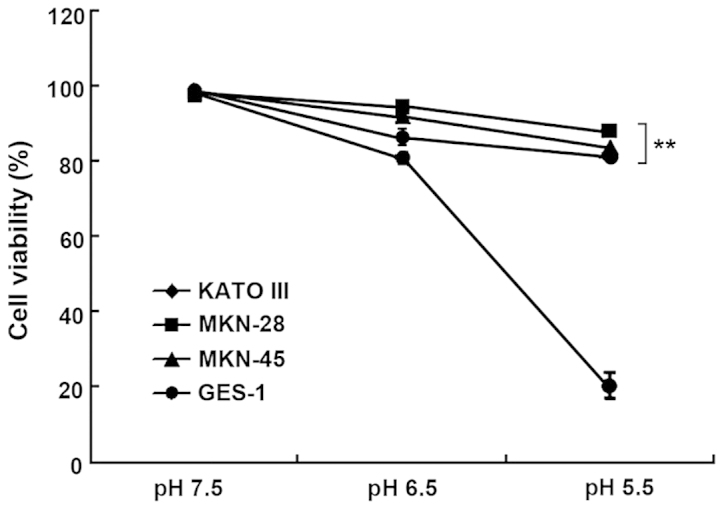
Effects of acidity of culture medium on cell viability. Human gastric cancer cell lines, KATO III, MKN-28 and MKN-45, and the non-cancer cell line, GES-1, were cultured in media at various pH levels (7.5, 6.5 and 5.5) for 24 h. The cells were then harvested and stained with trypan blue. The cell viability was assessed by dye exclusion assay. The gastric cancer cells were highly tolerant of acidic culture media compared with the non-cancer cells, whose viability significantly decreased at a lower pH value of pH 5.5. ^**^P<0.01 vs. the cell viability of the non-cancer cell line, GES-1.

To investigate whether gastric cancer cells exhibited better resistance to the acidic microenvironment, three gastric cancer cell lines, KATO III, MKN-28 and MKN-45, and one non-cancer cell line, GES-1, were cultured in media at pH values of 7.5, 6.5 and 5.5. As shown in Fig. 1, the gastric cancer cells were more tolerant to acidity, whereas the viability of the non-cancer cells significantly decreased in lower pH conditions (P<0.01). The viability of the GES-1 cells rapidly declined to 20.30±4.05%, while the gastric cancer cell lines retained high viability at pH 5.5, suggesting that gastric cancer cells adapt better to acidic conditions than non-cancer cells.

### Rabeprazole exerts a strong antiproliferative effect on gastric cancer cells under acidic conditions

**Figure 2:**
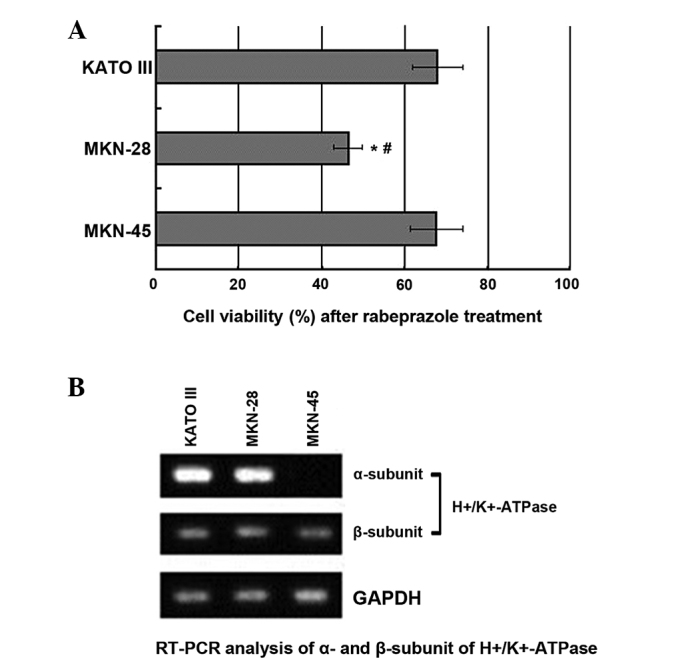
Decline in cancer cell viability induced by rabeprazole and expression level analysis of α - and β-subunits of hydrogen/potassium adenosine triphosphatase (H^+^/K^+^-ATPase) in gastric cancer cells. (A) Rabeprazole attenuated the cell viability of the human gastric cancer cells. Following treatment with 0.2 mM of the proton pump inhibitor rabeprazole for 16 h, the cell viability of the MKN-28 cells significantly decreased compared with the KATO III and MKN-45 cells, respectively. ^*^P<0.05 vs. the viability of the KATO III cells. ^#^P<0.05 vs. the viability of the MKN-45 cells. (B) Expression level analysis of α - and β-subunits of H^+^/K^+^-ATPase in the gastric cancer cells. The α-subunit of H^+^/K^+^-ATPase was strongly expressed in the KATO III and MKN-28 cells, while being weakly expressed in the MKN-45 cells. The β-subunit of H^+^/K^+^-ATPase was equally expressed in the tested cancer cells. Glyceraldehyde 3-phosphate dehydrogenase (GAPDH) was used as an internal control to verify equal amounts of total cDNA in each sample. RT-PCR, reverse transcription polymerase chain reaction

As aforementioned, the gastric cancer cells sustained high viability when cultured in acidic media. The increased proton efflux of cancer cells may play a protective role in maintaining their viability in adverse microenvironments. Rabeprazole was administrated to three gastric cancer cell lines, KATO III, MKN-28 and MKN-45, at a dosage of 0.2 mM for 16 h. The viability of these cells was determined by a trypan blue exclusion assay. Although rabeprazole treatment resulted in the attenuation of viability in all cancer cell lines tested (Fig. 2A), the ability of rabeprazole to induce cell death differed considerably between the cell lines. The viability of the MKN-28 cells was significantly lower than that of the KATO III and MKN-45 cells (P<0.05). In addition to changes in viability, the expression level of H^+^/K^+^-ATPase, which is widely acknowledged as the target of rabeprazole, was examined. As Fig. 2B shows, the α-subunit of H^+^/K^+^-ATPase was highly expressed in the KATO III and MKN-28 cells, while being weakly expressed in the MKN-45 cells. However, the β-subunit of H^+^/K^+^-ATPase was equally expressed in all the cancer cell lines, based on the similar expression level of total proteins controlled by GAPDH. These results suggest that, in terms of rabeprazole treatment, the ability to induce cell death was not clearly correlated with the expression level of H^+^/K^+^-ATPase.

### Rabeprazole attenuates cell viability via induction of apoptotic cell death in gastric cancer cells

**Figure 3:**
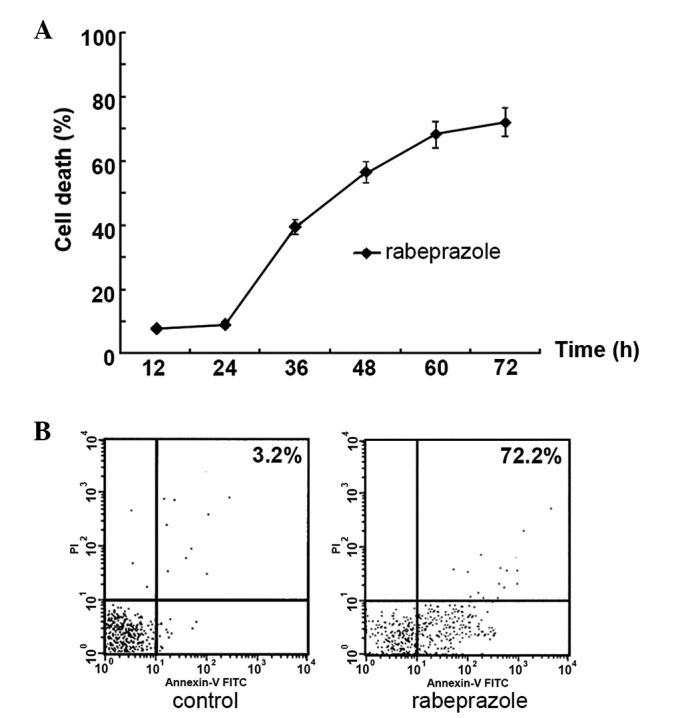
Rabeprazole treatment induces apoptotic cell death in gastric cancer AGS cells. (A) Kinetics of apoptotic cell death (%) induced by rabeprazole. Exposure to 0.2 mM rabeprazole resulted in significant apoptosis in the AGS cells after 72 h. (B) Annexin V-FITC)/PI double staining fluorescence-activated cell sorting analysis. Cells were harvested, stained with annexin V-FITC/PI, and analyzed by flow cytometry following treatment with rabeprazole. Administration of rabeprazole to the AGS cells markedly increased the apoptosis rate, reaching 72.21±3.24% compared with 3.20±0.26% in the control group (P<0.01). FITC, fluorescein isothiocyanate; PI, propidium iodide.

The results indicated that the treatment of rabeprazole led to increasing cell death in a time-dependent manner (Fig. 3A). To determine whether rabeprazole affected gastric cancer cell viability via the induction of apoptosis, the gastric cancer AGS cells were treated with 0.2 mM rabeprazole for 24 h. Following annexin V-FITC/PI staining, gastric cancer cell apoptosis was detected by flow cytometry. Rabeprazole treatment induced apoptosis in the treated cells (Fig. 3B). The AGS cells displayed significant apoptosis following treatment with rabeprazole for 72 h compared with the control group (P<0.01). The administration of rabeprazole resulted in an increased rate of apoptosis over time, reaching 72.21±3.24% subsequent to treatment for 72 h. Simultaneous staining of cells with annexin V-FITC/PI distinguished between the intact cells, early apoptosis, late apoptosis and cell death. These results indicated that exposure to rabeprazole mainly led to early apoptosis in the AGS cells (Fig. 3B).

### Rabeprazole inhibits the phosphorylation of ERK1/2 in human gastric cancer cells

**Figure 4:**
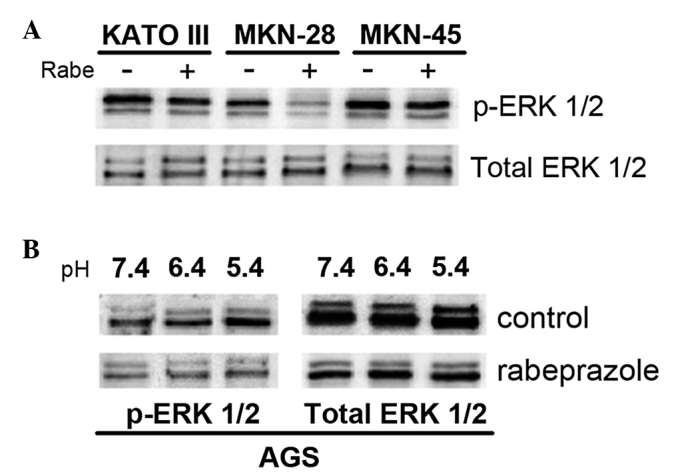
Inhibition of p-ERK 1/2 by rabeprazole (rabe). (A) Three gastric cancer cell lines (KATO III, MKN-28 and MKN-45) were cultured in acidic culture media (pH 5.4) for 2 h. Pretreatment with 0.2 mM rabeprazole for 2 h led to strong inhibition of ERK 1/2 phosphorylation in the MKN-28 cells, but a similar effect was not observed in the KATO III and MKN-45 cells. (B) The AGS cells were pretreated with 0.2 mM rabeprazole for 2 h, and subsequently incubated in culture media with various pH levels (7.4, 6.4 and 5.4) for 2 h. Treatment with rabeprazole was able to suppress ERK 1/2 phosphorylation in the AGS cells. P-ERK, phosphorylated extracellular signal-regulated protein kinase 1/2.

**Figure 5:**
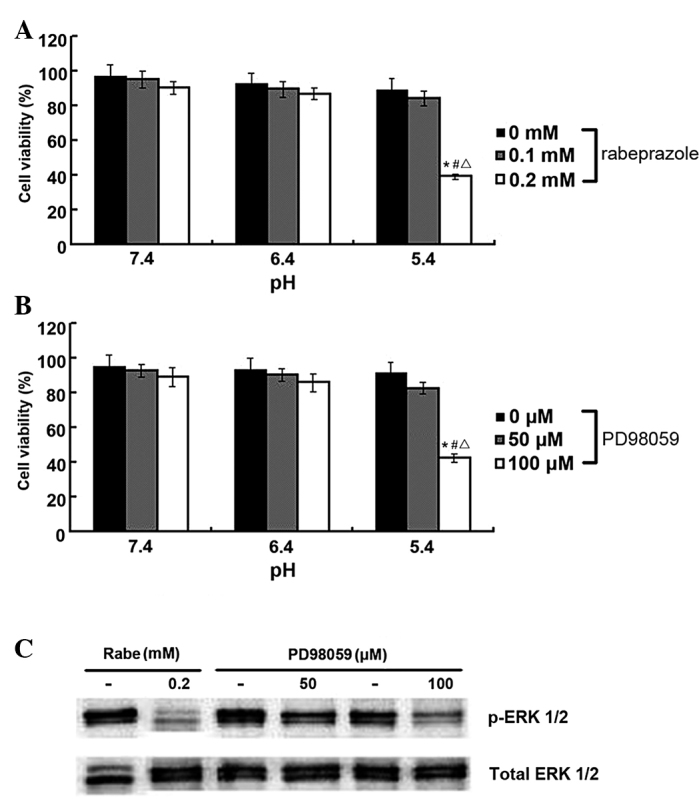
Effect of suppression of ERK 1/2 on cell viability. Following treatment with proton pump inhibitor rabeprazole or PD98059 (a potent ERK 1/2 inhibitor) for 2 h, the AGS cells were further cultured at various pH levels (pH 7.4, 6.4 or 5.4) for 16 h. (A) Administration of rabeprazole to the AGS cells at a dosage of 0.2 mM resulted in a marked attenuation in cancer cell viability at pH 5.4. ^*^P<0.05 vs. 0 mM rabeprazole treatment at pH 5.4. ^#^P<0.05 vs. 0.1 mM rabeprazole treatment at pH 5.4. ^Δ^P<0.05 vs. 0.2 mM rabeprazole treatment at pH 7.4 or 6.4. (B) Pretreatment with 100 μM PD98059 significantly reduced the cell viability at pH 5.4. ^*^P<0.05 vs. 0 μM PD98059 treatment at pH 5.4. ^#^P<0.05 vs. 50 μM PD98059 treatment at pH 5.4. ^Δ^P<0.05 vs. 100 μM PD98059 treatment at pH 7.4 or 6.4. (C) PD98059 and rabeprazole were each able to efficaciously inhibit the phosphorylation of ERK 1/2 in the AGS cells. P-ERK, phosphorylated extracellular signal-regulated protein kinase 1/2; Rabe, rabeprazole

The human gastric cancer cells showed strong resistance to hypoxic and acidic environments, while the non-cancer cells could hardly tolerate such adverse conditions. It has been reported that ERK 1/2, a subgroup of the mitogen-activated protein kinases (MAPKs), may contribute to cancer cell survival within acidic environments (14). The present study therefore examined the effect of rabeprazole on signal transduction through the ERK 1/2 pathway, which regulates many cellular activities, particularly cell proliferation and apoptosis. The KATO III, MKN-28 and MKN-45 gastric cell lines were treated with 0.2 mM of the PPI rabeprazole at pH 5.4. Although the phosphorylation of ERK 1/2 in the MKN-28 cells was completely suppressed by the administration of rabeprazole, the same effect was not observed in the KATO III and MKN-45 cells (Fig. 4A). The MKN-28 cells also displayed significant attenuation in cell viability compared with the KATO III and MKN-45 cells following rabeprazole treatment (Fig. 2A). These results indicated that the sensitivity to rabeprazole-induced apoptosis in gastric cancer cells may be correlated with the inhibitory efficacy of rabeprazole on ERK 1/2 phosphorylation. Furthermore, the study evaluated the inhibitory effect of rabeprazole on ERK 1/2 phosphorylation at various pH levels, including pH 7.4, 6.4 and 5.4. Treatment with rabeprazole suppressed ERK 1/2 phosphorylation in the AGS cells. However, ERK 1/2 activation increased as pH declined in the control cells (Fig. 4B). To determine whether the induction of apoptosis in the gastric cancer cells was due to the inhibitory actions of rabeprazole on ERK 1/2 phosphorylation, PD98059, a potent ERK 1/2 inhibitor, was administered to the AGS cells. As shown in Fig. 5B, the viability of the AGS cells significantly decreased following treatment with PD98059 at pH 5.4 (P<0.05). Similar antiproliferative effects were observed in the AGS cells within the rabeprazole treatment group (Fig. 5A). In addition, these results indicated that PD98059 and rabeprazole could each exert strong inhibitory effects on ERK 1/2 phosphorylation in the gastric cancer cells (Fig. 5C), thus suggesting that PD98059-induced cell death may follow a similar pattern of apoptotic cell death induced by rabeprazole. Consequently, it is conceivable that rabeprazole-induced inhibition of the phosphorylation of ERK 1/2 participates in the attenuation of cell viability and the induction of apoptosis in gastric cancer cells.

## Discussion

Current antineoplastic strategies are aimed at promoting the efficiency and specificity of therapies for gastric cancer. The present study indicated that rabeprazole, a second-generation PPI, could efficaciously induce apoptosis in human gastric cancer cells by blocking the intracellular proton extrusion, thus supporting the idea of PPIs as a potential treatment for gastric cancer (16,17). Highly proliferative cancer cells produce a large amount of H^+^ generated by aerobic glycolysis, lactic acid production and proton extrusion, leading to a lower extracellular pH level compared with normal tissues (18,19). As promising anticancer pro-drugs, PPIs require protonation in acidic conditions to become fully activated prior to acting on H^+^/K^+^-ATPase (20). Specific drug delivery to the acidic compartments and specific transformation into the active form within the acidic microenvironment are therefore permitted by this chemical property, thus inducing selective apoptosis in gastric cancer cells (21). Additionally, rabeprazole can be activated faster and has a higher accumulation even in the weakly acidic environment compared with other frequently used PPIs, owing to its relatively higher pKa (6,7,22,23). Therefore, it can be rapidly converted into its active form in acidic conditions within tumors and possibly serve as a novel approach, which is of high efficiency and specificity, for antineoplastic therapy.

As a potent PPI, rabeprazole is able to inactivate H^+^/K^+^-ATPase, causing a rapid and sustained inhibition of the gastric acidification (24). The present results suggested that rabeprazole is able to exert an antineoplastic effect on gastric cancer cells via the induction of apoptosis, and that the molecular mechanism of this effect involves its inhibitory action on the phosphorylation of ERK 1/2 in human gastric cancer cells. It is therefore possible that rabeprazole can be used as an adjuvant anticancer agent.

Despite the specific antiproliferative action of rabeprazole on cancer cells, a similar effect was not observed in the non-cancer cells in the present study. This discrepancy can be attributed to the induction of anti-apoptotic molecules HSP70 and HSP27 (13). Consequently, rabeprazole can induce apoptotic cell death in human gastric cancer cells without exerting any potential side-effects on non-cancer cells. Overall, these results suggest that rabeprazole may specifically target gastric cancer cells within acidic microenvironments, generated by upregulated glycolysis, without significant systemic toxicity.

In the present study, the gastric PPI rabeprazole attenuated the viability of three gastric cancer cell lines, KATO III, MKN-28 and MKN-45. However, the efficacy of this inhibitory action differed between the three cancer cell lines. The viability of the MKN-28 cells was markedly reduced when compared with that of the KATO III and MKN-45 cells. The present results also demonstrated that administration of rabeprazole induced significant apoptosis of the AGS cells in a time-dependent manner and caused early apoptotic cell death. Since the inhibition of intracellular proton efflux by PPI plays a participating role in the induction of apoptosis in gastric cancer cells, it is highly possible that the expression level of proton transporters determines the sensitivity to rabeprazole-induced apoptosis in anticancer therapy (14). However, the present study suggested that the ability of rabeprazole to induce cell death does not strongly correlate with the expression level of H^+^/K^+^-ATPase. Following treatment with rabeprazole, the viability of the MKN-45 cells was not as decreased as the viability of the MKN-28 cells, despite the strong expression of H^+^/K^+^-ATPase. This result indicates that, besides inhibitory effects on H^+^/K^+^-ATPase, rabeprazole may affect the activities of the other proton transporters that contribute to cell function and survival. In cancer cells, various intra- and extracellular pH regulatory mechanisms operate simultaneously to maintain cellular pH homeostasis. Among these mechanisms, vacuolar H^+^-ATPase (V-ATPase) is another crucial proton transporter due to its distinctive role in determining the acidification of the tumor microenvironment and therefore, the elimination of toxic molecules, such as H^+^ or reactive oxygen species (25). V-ATPase participates in the proton efflux by extruding protons into the extracellular environment or lumen of particular membrane-bound organelles to avoid the accumulation of H^+^ within the cytosol, resulting in extracellular acidification (26–28). Therefore, V-ATPase may also participate in the enhancement of tumor cell viability under hypoxic and acidic conditions via the extrusion of protons, providing another potential approach for an antineoplastic strategy.

Previous studies have shown that the MAPK pathway may contribute to cancer cell apoptosis and proliferation (29). As a subgroup of the MAPKs, ERK 1/2 plays a crucial role in the regulation of cell proliferation (30). In the present study, pretreatment with rabeprazole inhibited the ERK 1/2 phosphorylation and the viability of these cells, indicating that the inhibition of ERK 1/2 phosphorylation may result in the induction of apoptosis in gastric cancer cells. Moreover, the sensitivity to rabeprazole-induced apoptotic cell death in the gastric cancer cells appeared to be correlated with the inhibitory efficacy of rabeprazole on ERK 1/2 phosphorylation. ERK 1/2 phosphorylation was completely inhibited by rabeprazole in the MKN-28 cells, consistent with its high sensitivity to rabeprazole-induced apoptosis. However, significant ERK 1/2 phosphorylation inhibition was not observed in either the KATO III or MKN-45 cells, whose cell viability decreased slightly compared with the MKN-28 cells treated within the same protocol. Furthermore, the rabeprazole-induced inhibition of ERK 1/2 phosphorylation plays a participating role in the attenuation of cancer cell survival under acidic conditions. The potent ERK 1/2 inhibitor, PD98059, also exerted an inhibitory effect on ERK 1/2 phosphorylation and simultaneously induced apoptosis in the gastric cancer cells. Additionally, treatment with rabeprazole led to similar effects in the same cancer cell lines. Thus, it is conceivable that rabeprazole can induce apoptosis in human gastric cancer cells through inhibition of the phosphorylation of ERK 1/2.

In summary, the present results demonstrated that rabeprazole can attenuate cell viability via the induction of apoptosis in human gastric cancer cells. The mechanism underlying this antiproliferative effect of rabeprazole involves the inhibition of ERK 1/2 phosphorylation in gastric cancer cells. Thus, this study provides mechanistic insight and supports the use of rabeprazole as a promising agent for an anticancer strategy. However, the potential antiproliferative effects of the agent *in vivo* require investigation in the future.
